# Comparing the brain–behaviour relationship in acute and chronic stroke aphasia

**DOI:** 10.1093/braincomms/fcad014

**Published:** 2023-03-29

**Authors:** Natalie Busby, Argye E Hillis, Lisa Bunker, Chis Rorden, Roger Newman-Norlund, Leo Bonilha, Erin Meier, Emily Goldberg, Gregory Hickok, Grigori Yourganov, Julius Fridriksson

**Affiliations:** Department of Communication Sciences and Disorders, University of South Carolina, Columbia, SC 29209, USA; Department of Neurology, Johns Hopkins School of Medicine, Baltimore, MA 21287, USA; Department of Cognitive Science, Johns Hopkins University, Baltimore, MA 21218, USA; Department of Physical Medicine and Rehabilitation, Johns Hopkins University, Baltimore, MA 21287, USA; Department of Neurology, Johns Hopkins School of Medicine, Baltimore, MA 21287, USA; Department of Psychology, University of South Carolina, Columbia, SC 29208, USA; Department of Communication Sciences and Disorders, University of South Carolina, Columbia, SC 29209, USA; Department of Neurology, Emory University, Atlanta, GA 30322, USA; Department of Neurology, Johns Hopkins School of Medicine, Baltimore, MA 21287, USA; Department of Communication Sciences and Disorders, Northeastern University, Boston, MA 02115, USA; Department of Neurology, Johns Hopkins School of Medicine, Baltimore, MA 21287, USA; Department of Communication Disorders, University of Pittsburgh, Pittsburgh, PA 15260, USA; Department of Cognitive Sciences, University of California, Irvine, CA 92697, USA; Department of Language Science, University of California, Irvine, CA 92697, USA; Advanced Computing and Data Science, Cyberinfrastructure and Technology Integration, Clemson University, Clemson, SC 29634, USA; Department of Communication Sciences and Disorders, University of South Carolina, Columbia, SC 29209, USA

**Keywords:** aphasia, acute, chronic, stroke, lesion

## Abstract

In stroke aphasia, lesion volume is typically associated with aphasia severity. Although this relationship is likely present throughout recovery, different factors may affect lesion volume and behaviour early into recovery (acute) and in the later stages of recovery (chronic). Therefore, studies typically separate patients into two groups (acute/chronic), and this is often accompanied with arguments for and against using data from acute stroke patients over chronic. However, no comprehensive studies have provided strong evidence of whether the lesion–behaviour relationship early in recovery is comparable to later in the recovery trajectory. To that end, we investigated two aims: (i) whether lesion data from acute and chronic patients yield similar results in region-based lesion-symptom mapping analyses and (ii) if models based on one timepoint accurately predict the other. Lesions and aphasia severity scores from acute (*N* = 63) and chronic (*N* = 109) stroke survivors with aphasia were entered into separate univariate region-based lesion-symptom mapping analyses. A support vector regression model was trained on lesion data from either the acute or chronic data set to give an estimate of aphasia severity. Four model-based analyses were conducted: trained on acute/chronic using leave-one-out, tested on left-out behaviour or trained on acute/chronic to predict the other timepoint. Region-based lesion-symptom mapping analyses identified similar but not identical regions in both timepoints. All four models revealed positive correlations between actual and predicted Western Aphasia Battery-Revised aphasia-quotient scores. Lesion-to-behaviour predictions were almost equivalent when comparing within versus across stroke stage, despite differing lesion size/locations and distributions of aphasia severity between stroke timepoints. This suggests that research investigating the brain–behaviour relationship including subsets of patients from only one timepoint may also be applicable at other timepoints, although it is important to note that these comparable findings may only be seen using broad measures such as aphasia severity, rather than those aimed at identifying more specific deficits. Subtle differences found between timepoints may also be useful in understanding the nature of lesion volume and aphasia severity over time. Stronger correlations found when predicting acute behaviour (e.g. predicting acute: *r* = 0.6888, *P* < 0.001, predicting chronic *r* = 0.5014, *P* < 0.001) suggest that the acute lesion/perfusion patterns more accurately capture the critical changes in underlying vascular territories. Differences in critical brain regions between timepoints may shed light on recovery patterns. Future studies could focus on a longitudinal design to compare acute and chronic patients in a more controlled manner.

## Teaser

No comprehensive studies have compared the lesion–behaviour relationship throughout stroke aphasia recovery. Busby *et al*. report that lesion-to-behaviour predictions were almost equivalent when comparing within versus across stroke stage, despite differing lesion factors and aphasia severity. Differences between timepoints may aid the understanding of recovery patterns.

## Graphical Abstract

**Graphical abstract:**
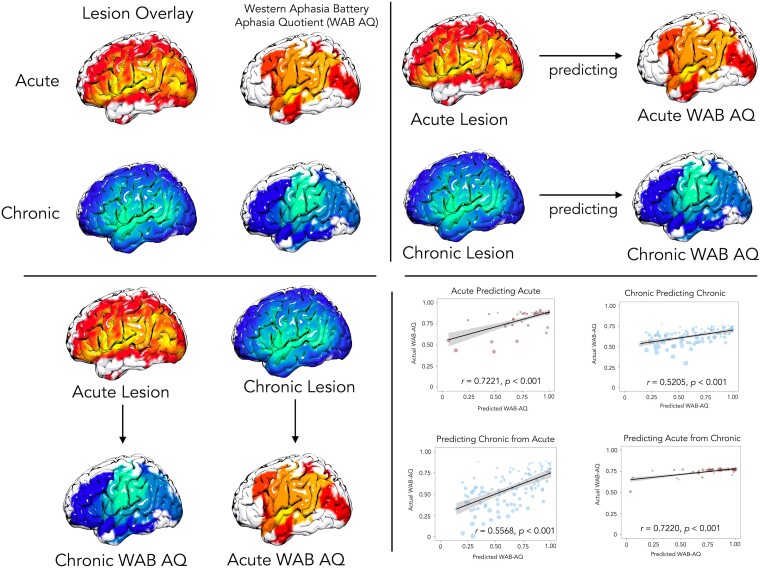

## Introduction

Ever since Broca^1^ and Wernicke^2^ first linked localized cortical structural damage to specific types of language impairment, exploration of the relationship between cortical integrity and behavioural deficits has been an integral part of understanding the relationship between the brain and behaviour. Stroke survivors provide unique insights into this relationship as nearly one-half of individuals experience aphasia as a consequence of stroke.^3^ Moving beyond the relatively simplistic and coarse-grained brain–behaviour analyses adopted in early studies of language and stroke, modern techniques such as voxel-based lesion-symptom mapping (VLSM), first used by Bates *et al.*,^4^ have become the cornerstone of modern-day investigations into the complex interplay between lesion size, lesion location and aphasia severity. Since then, many studies have used this technique to explore post-stroke deficits^5-12^ both from a clinical and from an experimental perspective in both the acute and the chronic stages of recovery. Unsurprisingly, in almost all cases, lesion size and location are important determinants of language deficit and therefore of aphasia subtype.^13-16^ Although stroke recovery is a continuous process, historically, researchers specializing in acute and chronic stroke have taken a conservative approach when considering lesion-based prognostic models of aphasia severity, treating acute and chronic populations separately. Indeed, to date, no single study has directly compared lesion-based models of recovery across both ends of the recovery trajectory (i.e. acute versus chronic), even though such a comparison has clear implications for clinicians aspiring to improve prognostic models of aphasia severity.

It is a common view that the rate of spontaneous recovery from aphasia in stroke survivors is the highest during the first 3 months^17-19^ and typically plateaus by 6 months post-stroke.^19-22^ Behaviour in the acute phase is known to fluctuate within individuals,^23^ and some people who initially present with aphasia may not display language deficits in the chronic stages. The unpredictable nature of recovery in the acute stage has been used to justify investigations of the brain–behaviour relationship post 6 months (chronic) where researchers typically have better control over potentially confounding variables likely to increase variability in language production and comprehension. Conversely, a benefit of examining brain–behaviour relationships early in the recovery trajectory (acute) is that recovery has not yet been influenced by factors like differential therapy types/amounts, and individuals have not yet developed cognitive strategies to compensate for deficits that may affect both the brain and behaviour.^21,24-29^ Finally, analyses early in recovery may also minimize the likelihood of any morphological changes that may have occurred, such as changes in the shape, location and quantity of brain tissue damaged in the later stages of recovery.^24,30^ Morphological changes may include structural distortions, sulcal or ventricle widening.^24^ As the brain has not had the time to functionally reorganize, VLSM studies conducted in the acute stages of recovery may capture the relationship between the brain and behaviour more accurately.^24^

While there are certainly advantages to studying stroke early in recovery, the difficulty in controlling potentially confounding variables has led many researchers to view chronic models as superior for investigating the brain–behaviour relationship in stroke. For example, testing in the chronic phase reduces the impact of immediate and uncontrollable consequences of the stroke, such as inflammation and swelling,^24,31^ along with possible reorganization between the acute and subacute phases.^32-41^ Furthermore, early testing often occurs in a stressful setting (e.g. a hospital) which may affect behavioural results, and patients may be taking multiple medications that vary as a function of the number and severity of immediate post-stroke sequalae. There may also be alterations in blood flow and metabolic activity associated with recovery and repair processes (reperfusion of tissue and resolution of diaschisis)^42,43^ unique to the first few weeks after stroke. Conversely, testing in the chronic phase is more often associated with a stable testing environment and a stabilization of blood flow and metabolic activity. In addition to these concerns, historically, there were several practical reasons why studies were seldom conducted early into recovery. For example, T_1_-weighted images may not show the full extent of structural damage in the acute phase; therefore, other imaging modalities may be necessary to identify damaged tissue accurately. More recently, increased availability of MRI scans with clinically feasible acquisition sequences has led to greater opportunities to study patient populations immediately post-stroke.^34-36,41,44,45^ However, all MRI scans reflect a tradeoff between scan time and quality, and people in the later stages of recovery are typically more stable and able to tolerate longer sessions relative to early recovery.

Although studies typically include only acute or chronic models of stroke, there has been no general consensus concerning incorporating different stages of recovery into one model, or whether there are substantial differences in the brain-to-behaviour relationship between early and late stages of recovery. Some studies have attempted to incorporate both acute and chronic patients into a single model, although directly comparing the two has never been a primary aim. Hillis *et al*.^46^ demonstrated that auditory word comprehension deficits occur if the left posterior superior temporal gyrus (pSTG) abruptly becomes damaged. Still, in some cases, the brain can adapt to this change, and over time, other regions may assume the function of damaged regions. This network-level inhibition and functional reorganization may explain some of the differences noted between acute and chronic studies throughout the literature but does not directly address the question of how comparable the relationship between the lesion and behavioural deficits is at these timepoints. In another study that attempted to understand stroke in multiple stages of recovery, Karnath and Rennig^30^ directly compared combinations of acute and chronic behaviour and lesion information using motor system representation as a proof-of-concept design. Although they concluded that a combination of acute imaging and acute behaviour was most predictive of the underlying neural substrates (i.e. damage to the corticospinal tract), some of the relationships they tested (e.g. using acute imaging with chronic behaviour) were neither clinically relevant nor simple to interpret.

Despite these arguments for and against using data from different stages of stroke recovery, to date, no study has used a brain–behaviour approach to directly compare the relationship between lesion size/location and behavioural impairment at the acute and chronic stages of aphasia recovery. Therefore, our goal was to use a cross-sectional study design to compare machine-learning-based predictions of language impairment generated at different stages of stroke recovery trajectory. This may be useful for medical professionals interested in prognosis and long-term treatment of stroke, and for integration of previous research which captured behavioural deficits from only one stage of recovery. To that end, we investigated two main aims: (i) whether the relationship between lesion load and behavioural impairment (aphasia severity) is similar in acute and chronic patients [i.e. do region-based lesion-symptom mapping (RLSM) analyses yield similar results] and (ii) if models of aphasia severity based on acute lesion data are able to accurately predict aphasia severity in a chronic dataset, and *vice versa*. We hypothesize that in both acute and chronic patients, larger lesion volume will be associated with more severe aphasia [i.e. lower Western Aphasia Battery-Revised Aphasia Quotient (WAB-R AQ) score] and that similar regions (those found in the dual stream models of language) will predict aphasia severity. We also hypothesize that within sample predictions (i.e. acute predicting acute or chronic predicting chronic) will be more accurate than across sample predictions (e.g. acute predicting chronic or chronic predicting acute).

## Materials and methods

### Acute participants

**Figure 1:**
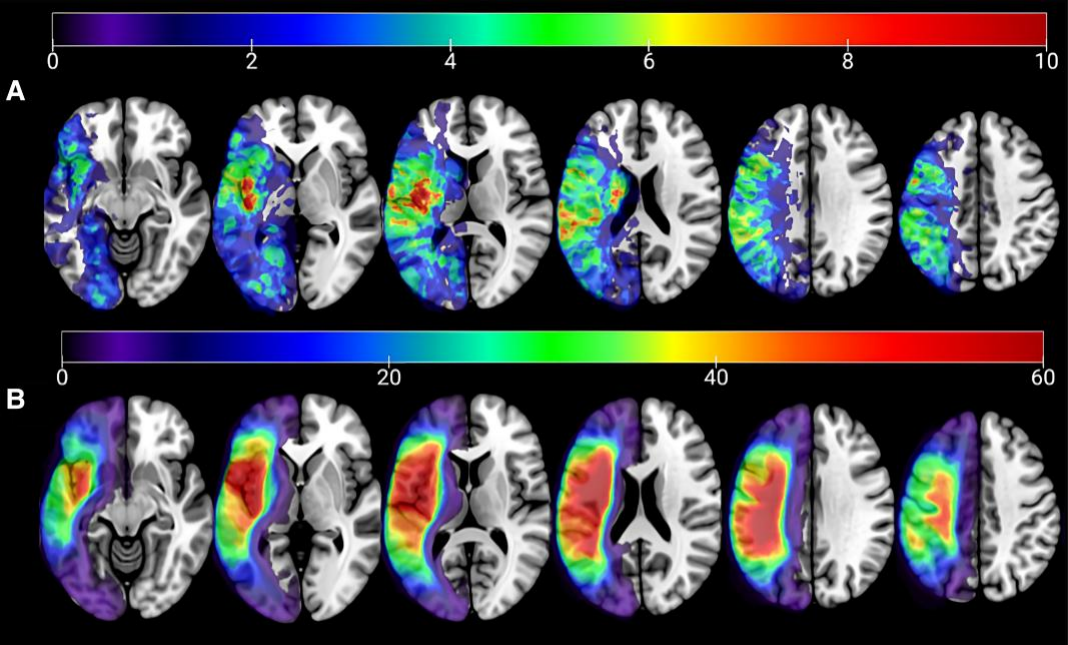
**Lesion overlay map.** (**A**) Acute participants and (**B**) chronic participants. Warmer colours indicate greater lesion overlap (i.e. more patients have damage in this region). The colour bar indicates the minimum number of people who have damage in that region.

Participants were enrolled at Johns Hopkins Hospital in the first 48 h following left hemisphere ischaemic stroke and MRI scans and behavioural testing were administered no more than 10 days post-onset. For every individual, MRI scans were collected before behavioural testing. For the majority of patients (45) scans were either on the same day as behavioural testing or in the 48 h prior. Due to some patients being transferred from other hospitals, 13 individuals had an MRI scan 3–6 days before behavioural testing (3 days: 7 individuals, 4 days: 6 individuals, 6 days: 5 individuals). The following inclusion criteria were utilized: (i) no impaired level of consciousness or ongoing sedation; (ii) no lack of premorbid competence in English; (iii) no previous neurological disease; (iv) able to be scanned (i.e. no implanted ferrous metal, no claustrophobia, etc.) and (v) no stroke within the cerebellum. Although there was no explicit exclusion of individuals with damage to the right hemisphere, participants within this study had lesions restricted to the left hemisphere (see Fig. 1). Trained research assistants or American Speech and Language Association (ASHA)-certified speech-language pathologists (SLPs) with experience working with individuals with aphasia administered all assessments. The acute sample comprised *N* = 63 individuals (34 females) with a mean age of 60.0 years [standard deviation (SD) = 12.3] at the time of stroke (see Table 1). Participants were an average of 2.6 (SD = 2.3) days post-stroke.

**Table 1:** Demographic information of participants

	Acute	Chronic
Age at stroke (years)		
Mean	60.0	56.4
SD	12.3	11.5
Range	29.0–91.0	27.0–79.0
Age at testing (years)		
Mean	60.0	60.8
SD	12.3	10.8
Range	29.0–91.0	29.2–80.1
Days post-stroke		
Mean	2.6	1672.9
SD	2.3	1687.2
Range	0–10	359–7347
Education		
Mean	14.3	15.5
SD	3.1	2.3
Range	4–20	12–20
Lesion size (number of voxels)		
Mean	22 510.4	118 808.6
SD	42 812.8	94 864.7
Range	124–268 332	117–467 458
WAB-R AQ		
Mean	80.0	62.9
SD	23.5	24.7
Range	3.5–100	14.5–100
Spontaneous speech		
Mean	15.2	12.1
SD	5.1	5.2
Range	0–20	3–20
Auditory verbal comprehension		
Mean	172.3	157.9
SD	46.3	37.3
Range	9–200	51–200
Repetition		
Mean	83.4	56.1
SD	25.3	30.5
Range	0–100	1–100
Naming		
Mean	78.3	58.6
SD	27.3	31.2
Range	0–100	1–100

### Chronic participants

Chronic participant data were extracted from a database collected at the Center for the Study of Aphasia Recovery at the University of South Carolina and the Medical University of South Carolina. All chronic participant testing took place at research laboratories at the University of South Carolina and the Medical University of South Carolina. ASHA-certified SLPs with experience working with individuals with aphasia administered all assessments. Both behavioural testing and MRI scans were conducted on the same day.

The following inclusion criteria were utilized: (i) between the ages of 21 and 80; (ii) chronic aphasia (≥12 months post-stroke) due to left-hemispheres stroke; (iii) no severely limited output [measured by a Western Aphasia Battery-Revised (WAB-R) spontaneous speech rating scale score of 0–1], severely limited auditory comprehension (WAB-R auditory comprehension rating scale score of 0–1) and (iv) no accompanying psychiatric or neurological disorders, or a bilateral or cerebellar stroke. Individuals with multiple strokes were eligible if all structural lesions were confined to the left hemisphere.

This sample comprised *N* = 109 individuals (68 females) with a mean age of 56.35 years (SD = 11.54) at the time of stroke, and a mean test age of 60.80 (SD = 10.75). Participants were an average of 53.08 (SD = 54.44) months post-stroke at the time of testing (see Table 1 for full demographic information).

### Combined participants

All study procedures have been approved by Institutional Review Boards at each University, and all participants provided written informed consent to all study procedures. These data represent a convenience sample based on recruitment through advertising, referrals from neurologists and speech pathologists and active recruitment within support/aphasia groups. All participants completed a comprehensive battery of language tests, including the WAB-R.^47^

There was no significant difference between the acute and chronic patients in terms of age at stroke [acute; *M* = 60.00, SD = 12.3, chronic; *M* = 56.35, SD = 11.5, *t*(177) = 1.89, *P* = 0.334], age at the time of testing [acute; *M* = 60.00, SD = 12.3, chronic; *M* = 60.80, SD = 10.8, *t*(181) = −0.467, *P* = 0.124] or education [acute; *M* = 14.25, SD = 3.1, chronic; *M* = 15.49, SD = 2.3, *t*(173) = −2.93, *P* = 0.051].

### MRI data acquisition and preprocessing

Patients underwent high-resolution T_1_- and T_2_-weighted neuroimaging acquired on a Siemens Trio or Phillips 3 T scanner equipped with a 12-channel head coil using the following parameters: T_1_-weighted imaging utilized an MP-RAGE (magnetization-prepared rapid gradient-echo) sequence with 1 mm isotropic voxels, a 256 × 256 matrix size, a 9° flip angle and a 92-slice sequence with a repetition time = 2250 ms, inversion time = 925 ms and echo time = 4.11 ms. T_2_-weighted scans were acquired using a three-dimensional T_2_-weighted SPACE (Sampling Perfection with Application optimized Contrasts using different flip angle Evolution) sequence covering the whole head and with a resolution of 1 mm^3^ was used with a field of view = 256 × 256 mm, 160 sagittal slices, variable degree flip angle, repetition time = 3200 ms and echo time = 212 ms. Acute patients also underwent diffusion-weighted imaging (DWI) which was used to create apparent diffusion coefficient maps useful in identifying acutely infarcted areas. Normalization functions used to transform DWI into standard space were computed based on the B0 image (which does not show stroke-related abnormalities).

Lesions were drawn onto each subject’s DWI (acute patients) or T_2_-weighted image (for chronic patients). Acute lesions were drawn by trained researchers (authors L.B., E.M. and E.G.), all of whom were blinded to behavioural data. All acute lesion tracings were verified by an experienced researcher (author L.B.) to ensure consistency. Chronic lesions were drawn by an expert neurologist (author L.B.) or trained study staff members (authors R.N.-N., L.B., E.M. and E.G.), all of whom were blinded to the behavioural data. Enantiomorphic segmentation-normalization was then conducted using the *nii_preprocess* pipeline (https://github.com/neurolabusc/nii_preprocess)^48^, a set of MATLAB-based (R2017b, The MathWorks, Inc., Natick, MA, USA) scripts that leverage multiple best-of-breed programmes [SPM12; Functional Imaging Laboratory, Wellcome Trust Centre for Neuroimaging, Institute of Neurology, UCL (www.fil.ion.ucl.ac.uk/spm), FSL version 6.0.3,^49^ ASLtbx (https://cfn.upenn.edu/zewang/ASLtbx.php) and MRItrix (https://www.mrtrix.org/)] in order to normalize and process MRI data acquired from individuals with lesioned brains. These scripts utilized enantiomorphic normalization^43^ to create chimeric images (a.k.a. healed’ brains) in which the damaged portion of the left hemisphere was temporarily replaced with the mirror image of intact areas from the healthy right hemisphere (using SPM12’s Clinical Toolbox).^50^ SPM12’s unified segmentation-normalization^51^ warped this chimeric image to standard (MNI) space, and the resulting spatial transform was then applied to the native-space T_1_ scan as well as the native-space versions of the hand-drawn lesion map, T_2_ and DWI images. This additional step (enantiomorphic normalization) ensures that segmentation-normalization methods designed for intact brains do not incorrectly warp scans with large lesions to the left hemisphere and have been used successfully in multiple prior publications on stroke.^25,52-54^Figure 1 provides lesion overlay maps for each patient group.

### Lesion analyses

For the acute and chronic datasets, we conducted separate univariate RLSM analyses^55^ to predict WAB-R AQ scores from the percentage of damage to each region of interest (ROI). ROIs were defined using all regions in the Johns Hopkins University (JHU) atlas,^56^ analyses were performed using the NiiStat toolbox for MATLAB (https://www.nitrc.org/projects/niistat/). This method was chosen as it allowed the identification of brain regions which are related to aphasia severity. Subsequent separate analyses were also conducted for four WAB-R subscores; auditory verbal comprehension, naming and word-finding, repetition and spontaneous speech. Although these analyses included all participants, they were limited to ROIs that were damaged in at least 10% of participants (11 people for chronic set and 6 people for the acute set). This is to ensure that ROIs do not appear as significant predictors based on damage in only a small proportion of the participants. It should be noted that thresholding at 10% was an effort to balance maintaining a large sample size with reducing the oversize effects of one or two lesions, as in previous literature.^57-59^ To correct for multiple comparisons, we used permutation thresholding with 2000 permutations. We identified the ROIs where the damage was a significant predictor of the behavioural score (*P* < 0.05, corrected).

### Statistical analysis

As it is difficult to statistically compare outputs across timepoints using separate RLSM analyses, to address our second line of inquiry, we used linear support vector regression (SVR) models to predict the WAB-R AQ scores from percentage of damage for each JHU ROI. WAB-R AQ scores were normalized from the raw 0…100 values to 0…1.0 for all participants, regardless of group. Training the SVR model consisted of estimating the coefficients (beta weights) for each region (signifying the importance of that region in predicting the AQ scores), as well as a constant term (offset) so that a sum of weighted per cent damage for all regions plus the offset, gives an estimate of WAB-R AQ. When we use one set of participants for training and the other set for testing, we enter the per cent damaged for the test set into the model to get estimates of WAB-R AQ for the test set. Alternatively, within the same set of participants, we use a leave-one-out procedure, where we iteratively set aside one participant for testing and train the model on all remaining participants; this process is repeated until we obtain the estimated scores for all participants in the set (the leave-one-out procedure ensures that different data points are used to train and test the model). Four analyses were conducted:

1. The acute data set was processed using the leave-one-out procedure to train the model, and the model was then used to predict the WAB-R AQ scores for the left-out participants.
2. The chronic data set was processed using the leave-one-out procedure to train the model, and the model was then used to predict the WAB-R AQ scores for the left-out participants.
3. The acute data set was used to train the model, and the model was then used to predict WAB-R AQ scores in the chronic dataset.
4. The chronic data set was used to train the model, and the model was then used to predict WAB-R AQ scores in the acute dataset.

The prediction accuracy was computed as a Pearson correlation coefficient between the actual WAB-R AQ scores and predicted WAB-R AQ scores. For analyses (3) and (4), one model was created for the training set and then applied to the test set, so reported beta weights are from the single model. Analyses (1) and (2) were conducted using a leave-one-out approach; therefore, 62 models were calculated for the acute set and 108 for the chronic set (full sample size: 63 acute and 109 chronic). Reported beta weights for these analyses are averaged across these models. Beta weights are not *Z*-scored, so relative magnitude is relevant, but the absolute magnitude is difficult to interpret.

As overall lesion volume (rather than proportional damage to each ROI) is an easier-to-acquire predictor, we also repeated this analysis using lesion volume alone to predict WAB-R AQ scores.

### Ethical considerations

All participants gave informed consent for study participation in accordance with the Declaration of Helsinki.

## Results

**Figure 2:**
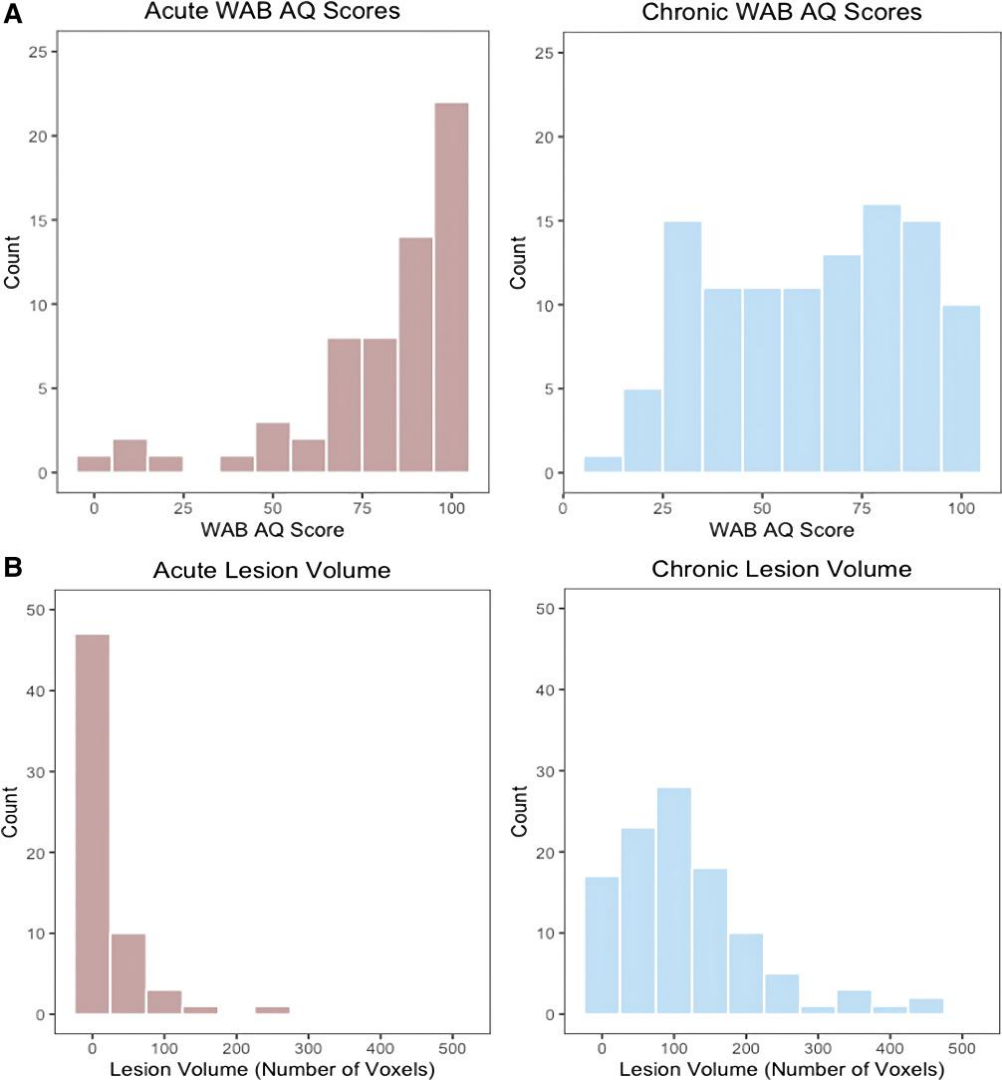
**Histograms of the distribution of aphasia severity and lesion volume.** WAB-R AQ scores (**A**) and lesion volume (**B**) are shown for the acute data set (left) and chronic data set (right). Lesion volume is displayed as the number of voxels, divided by 1000. *T*-tests revealed that there was no significant difference between acute and chronic WAB-R AQ score [acute; *M* = 80.01, SD = 23.46, chronic; *M* = 62.95, SD = 24.68, *t*(179) = −0.451, *P* = 0.059]. Acute patients (*M* = 22 510 voxels, SD = 42 813 voxels) had significantly smaller lesions than chronic patients [*M* = 118 809 voxels, SD = 94 865 voxels, *t*(168) = −7.556, *P* < 0.001].

There was no significant difference between acute and chronic WAB-R AQ scores [acute; *M* = 80.01, SD = 23.46, chronic; *M* = 62.95, SD = 24.68, *t*(179) = −0.451, *P* = 0.059]. However, the overall distribution of scores varied between the two: acute (median = 87.90, range = 3.5–100) versus chronic (median = 64.60, range = 14.5–100). Therefore, although both groups had a broad range of aphasia severities, there were more chronic participants with moderate–severe aphasia. See Fig. 2 for histograms of these distributions. Acute patients (*M* = 22 510 voxels, SD = 42 813 voxels) had significantly smaller lesions (in terms of the total number of voxels) than chronic patients (*M* = 118 809 voxels, SD = 94 865 voxels, *t*(168) = −7.556, *P* < 0.001). Figure 2 shows histograms of the distribution of lesion volume (number of voxels) for both data sets.

**Figure 3:**
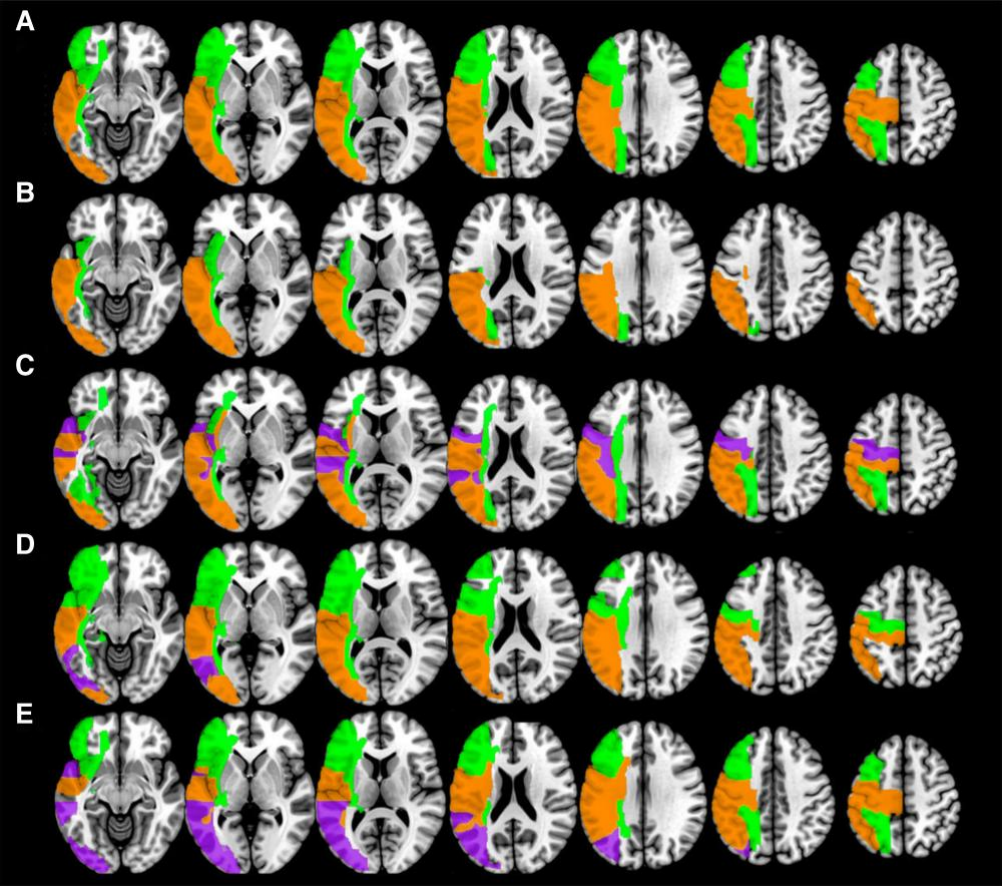
**RLSM results.** Regions which significantly predicted behavioural scores in the RLSM analysis. Orange is acute, green is chronic, and purple represents areas which overlap. (**A**) WAB-R AQ, (**B**) auditory verbal comprehension score, (**C**) naming and word-finding score, (**D**) repetition score and (**E**) spontaneous speech score.

A whole-brain RLSM analysis revealed that in both acute and chronic data sets, lesions to a specific subset of brain regions were significantly associated with reduced WAB-R AQ scores. These regions included areas associated with the dorsal language stream [superior longitudinal fasciculus (SLF), pre- and postcentral gyri, supramarginal gyrus (SMG), posterior corona radiata] and the ventral language stream [superior temporal gyrus (STG), pSTG, STG pole, middle temporal gyrus (MTG), posterior MTG (pMTG), inferior temporal gyrus, angular gyrus (AG), SMG], as well as inferior and middle occipital gyri [inferior occipital gyrus and middle occipital gyrus (MOG), respectively]. A further 19 regions were also associated with reduced WAB-R AQ scores only in the chronic data set (see Supplementary Table 1 for a complete list of ROIs that were significantly associated with WAB-R AQ and the four subscores). Figure 3 shows the ROIs that were significant in acute and chronic data, as well as overlap between these two analyses. To directly compare the overlap of results from the RLSM analysis, we calculated a dice similarity index (similarity coefficient = 0.738). However, it is important to note when interpreting these results, chronic participants had, on average, larger lesions than acute participants. Therefore, there may be some regions which are significant predictors of WAB-R AQ in the chronic RLSM analysis which were not lesioned in any of the acute participants.

**Figure 4:**
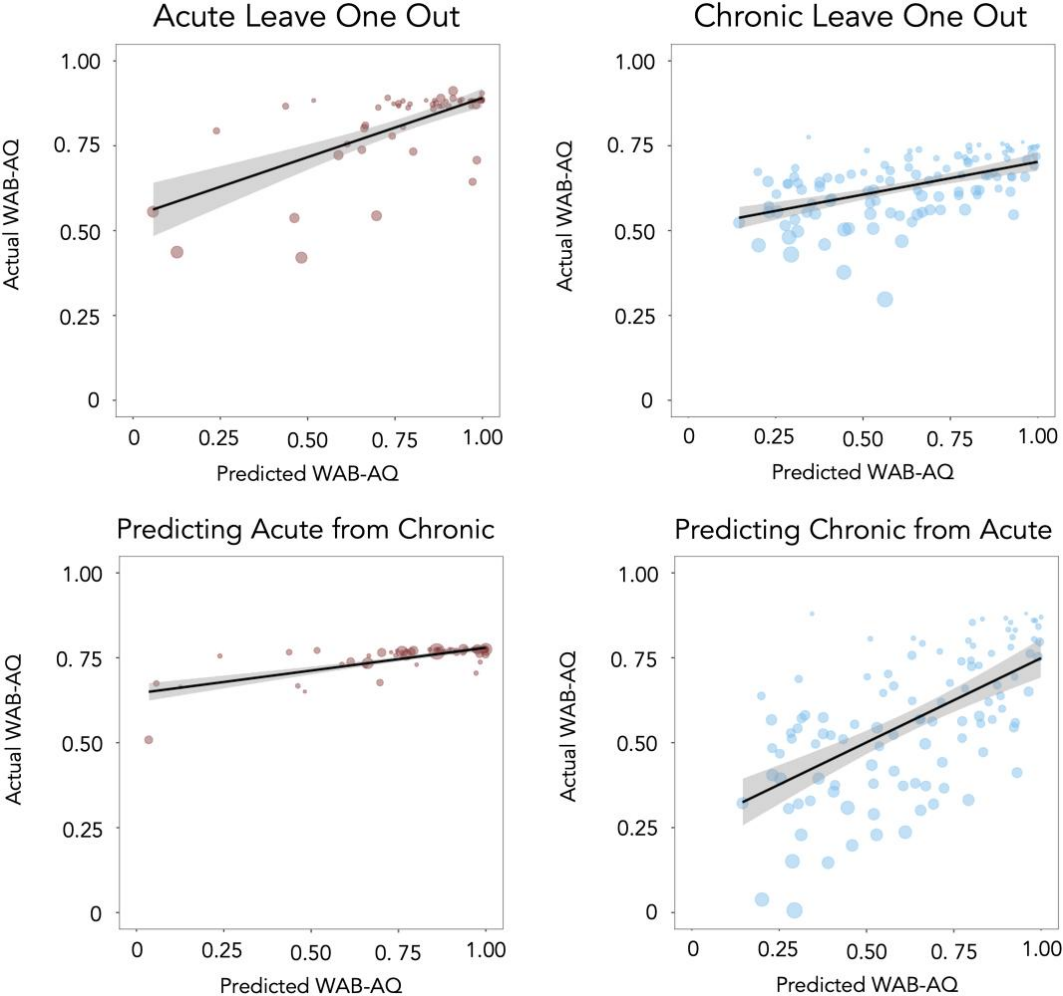
**Correlations between actual and predicted WAB-R AQ scores.** Acute leave-one-out model (*P* < 0.001) and chronic leave-one-out model (*P* < 0.001). Size of datapoints are proportional to lesion volume for each participant.

In addressing our second line of inquiry, the prediction accuracy of each model was estimated using Pearson’s correlation coefficient between actual and predicted WAB-R AQ scores. Results are as follows: positive correlations were found between actual WAB AQ scores and predicted WAB AQ scores for each analysis: predicting acute scores using leave-one-out (*r* = 0.7221, *P* < 0.001), predicting chronic scores using leave-one-out (*r* = 0.5205, *P* < 0.001), training on chronic, predicting acute scores (*r* = 0.7220, *P* < 0.001) and training on acute, predicting chronic scores (*r* = 0.5568, *P* < 0.001), as shown in Fig. 4. A two-tailed Fisher *r*-to-*z* transforms found that within-group training was not significantly different than between-group training for predicting acute scores (*z* = 0) or chronic scores (*z* = 0.37, *P* < 0.71). However, predicting acute scores was more accurate than predicting chronic scores for the within-group training (*z* = 2.07, *P* < 0.0192) with a numerical trend for the between-group training (*z* = 1.76, *P* < 0.0784).

**Figure 5:**
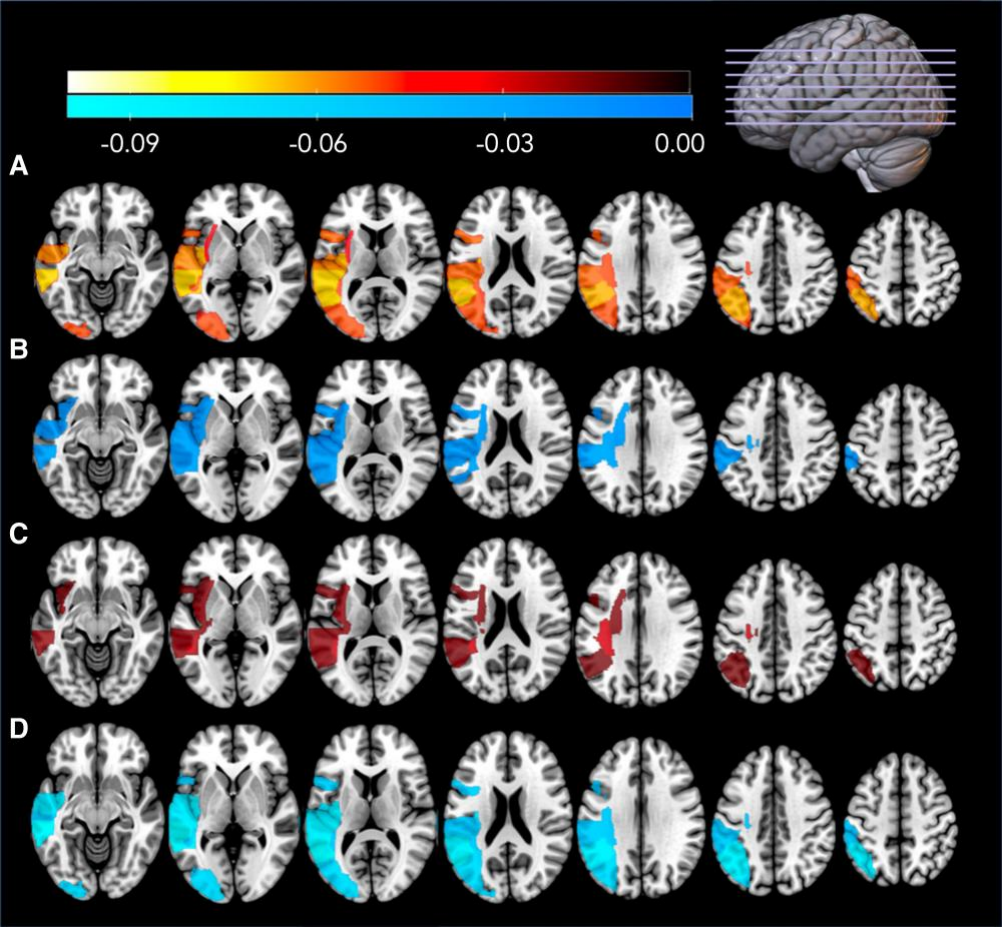
**Brain map of 10 lowest (most negative) beta maps for each analysis.** (**A**) Acute leave-one-out model, (**B**) chronic leave-one-out model, (**C**) predicting acute scores from chronic data and (**D**) predicting chronic scores from acute data.

Brain maps were calculated using the 10 lowest (most negative) beta weights for each analysis, see Fig. 5. As all individuals had left hemisphere lesions, all ROIs are in the left hemisphere. ROIs from the acute leave-one-out model (Fig. 5A) included the pMTG, pSTG, posterior insula, AG, STG, inferior frontal gyrus (IFG) pars opercularis, SMG, MOG, SLF and external capsule. The chronic leave-one-out model (Fig. 5B) included the SLF, posterior insula, pSTG, external capsule, pMTG, SMG, superior corona radiata, insular, STG and IFG pars opercularis. The lowest beta weights in the model predicting acute scores from chronic data (Fig. 5C) included the SLF, posterior insula, external capsule, pMTG, pSTG, retrolenticular portion of the internal capsule, superior corona radiata, IFG pars opercularis, AG and insula. Finally, the model predicting chronic scores from acute data (Fig. 5D) included pMTG, AG, posterior insula, pSTG, STG, MOG, IFG pars opercularis, MTG, SMG and SLF. A complete list of beta weights can be found in Supplementary Table 2.

Similar results were found for the prediction accuracy of each model when using lesion volume alone. Pearson’s correlation coefficients between actual and predicted WAB-R AQ scores are as follows: positive correlations were found between actual WAB-R AQ scores and predicted WAB-R AQ scores for each analysis: predicting acute scores using leave-one-out (*r* = 0.6888, *P* < 0.001), predicting chronic scores using leave-one-out (*r* = 0.5014, *P* < 0.001), training on chronic, predicting acute scores (*r* = 0.7242, *P* < 0.001) and training on acute, predicting chronic scores (*r* = 0.5274, *P* < 0.001). A two-tailed Fisher *r*-to-*z* transform found that within-group training was not significantly different than between-group training for predicting acute scores (*z* = 0.39, *P* = 0.6965) or chronic scores (*z* = 0.26, *P* = 0.7949). However, predicting acute scores was more accurate than predicting chronic scores for the between-group training (*z* = 2.04, *P* = 0.0414), with a numerical trend for the within-group training (*z* = 1.82, *P* = 0.0688).

## Discussion

Lesion size and location is an important determinant of aphasia severity at all stages of the stroke recovery trajectory; however, studies typically focus either on acute or chronic timepoints and rarely use both within the same analysis. While there are arguments in favour and against using one timepoint over the other, no comprehensive studies have provided strong evidence if the two are comparable in lesion-based analyses. Therefore, this study investigated whether lesion data and WAB-R AQ scores from acute and chronic patients yield similar results in RLSM and whether a model based on one set of patients (i.e. acute versus chronic) could predict scores in another independent sample of patients collected at a different timepoint (i.e. acute versus chronic). Our results demonstrated that a model based on an acute dataset can predict WAB-R AQ scores in a chronic dataset which is important both clinically and experimentally. From a clinical perspective, the ability to predict behaviour in the chronic stage based on data collected acutely is critical and could help to inform therapy and treatment approaches. This is particularly relevant given that stroke age is declining^60^ so it is likely people will live longer in the chronic stages of recovery. Experimentally, these data are in line with stroke recovery being a continuum and suggest that results from studies conducted in the acute phase of stroke can be applied to the chronic phase with a reasonable degree of accuracy. However, it should be noted that there is large variability which suggests that predictions are more accurate for some individuals compared to others. Therefore, these results may be more meaningful at a group level rather than a case-by-case level. Similar results in the converse analysis, which predicted acute outcomes using a model based on a chronic dataset, are less clinically relevant but are experimentally interesting as it suggests that this theory holds true, irrespective of brain changes or behavioural recovery. These lesion-to-behaviour predictions were almost equivalent when comparing within stroke stage (acute, chronic) versus across stroke stage and do not appear to get worse across the acute–chronic or chronic–acute boundaries. This evidence is compelling given that a between-subjects design was used, therefore there were critical methodological and statistical differences between our two groups that may have masked such a finding, e.g. that acute and chronic patients were recruited in different hospitals, lesions were identified using different modalities (drawn on diffusion-weighted images in acute, T_2_-weighted images in chronic) and WAB-R AQ scores were not evenly distributed across both datasets. Likewise, patients in the chronic stage of recovery typically had larger lesions (as shown in Fig. 1) and volunteered because they felt they had some long-term speech and language impairments, whereas the enrolment in the acute phase included all participants (some with no impairments, and others that would presumably resolve prior to the chronic stage). This indeed supports previous literature suggesting that the lesion is the greatest predictor of behaviour.^61-65^ As similar results were found when using both proportional damages to each ROI and lesion volume alone, overall lesion volume is likely driving this relationship. Although a longitudinal design would be an informative way to investigate differences in the brain–behaviour relationship at different timepoints along the recovery trajectory in a highly controlled way, a between-subjects design is also an important way to consider this question. As these patients in the acute and chronic stages of recovery have predominantly been studied separately, our results highlighting the similarities in the brain–behaviour relationship across the recovery trajectory means research conducted at one timepoint may also be relevant for the other timepoint. Furthermore, the accuracy of our predictions is very compelling, given the multiple differences between the two groups. This is clinically relevant because when seeing a patient at the acute stage, we might have some insight into their aphasia severity at the chronic stage, regardless of how well-matched factors are to chronic patients. However, future studies could utilize a longitudinal design to investigate the brain–behaviour relationship when participant factors are well-controlled.

Predictions of acute behaviour, irrespective of which timepoint was modelled, yielded higher correlations than those of chronic behaviour. It is possible that the acute lesion/perfusion pattern more accurately captures the critical changes in blood flow in the underlying vascular territories and supports the idea that the acute stage may capture the relationship between brain and behaviour more accurately. This could be due to a number of factors that influenced either brain structure/function or behaviour post-stroke, including, but not limited to: therapy and compensatory cognitive strategies may have improved behaviour, and resulted in morphological changes, both positive (e.g. functional reorganization) and negative (e.g. changes in perilesional integrity). Indeed, recent studies have shown improvement and declines in behaviour with therapy and compensatory strategies that can occur years beyond the stroke onset.^25,26,66-68^ The larger lesions found in the chronic phase likely reflect differing inclusion criteria but also support the idea of morphological changes occurring over time.^69^ It may be that the acute lesions are more experimentally informative about the brain–behaviour relationship, while the chronic lesions can give us more insight into recovery mechanisms. However, since different patient populations were used at each timepoint in this study, it is also likely that many of the acute patients who have smaller lesions may recover quicker and do not go on to have chronic aphasia. It should also be noted that there were fewer acute participants and less variability in behavioural scores at this timepoint, which may also have impacted results. This may have resulted in some restricted range issues, as there were less acute patients with moderate–severe aphasia compared to the chronic patients. Future studies could include a larger proportion of severe acute aphasia to determine whether predictions in the early stages of recovery are more accurate than the later stages when groups are more completely matched. Including other clinically relevant factors such as demographics (age, education, etc.) and acute severity may also explain some of the additional variances and, therefore, improve prediction accuracy. Factors such as these should be considered in future longitudinal comparisons of acute and chronic aphasia.

The differences in our samples may explain why similar, but not identical, results emerged from the RLSM analyses. All regions associated with WAB-R AQ scores in the acute patients were also identified in the chronic patients. This is a possible explanation for why acute predictions were high irrespective of whether acute or chronic data were initially modelled. However, the reverse was not true: in the RLSM analysis, several regions were only correlated in the chronic patients. One explanation is simply that lesion distributions differed between timepoints; chronic patients had larger lesions than acute, therefore it is unsurprising to find more regions associated with behavioural deficits in this population. However, many of the regions only found in chronic patients were white matter tracts (e.g. the internal and external capsules, anterior and superior corona radiata, IFOF [inferior fronto-occipital fasciculus], UF [uncinate fasciculus], etc.). Extensive degeneration in white matter integrity across the brain is often found post-stroke^70^ which likely explains why these regions are only found within the chronic cohort. It has also been suggested that small vessel disease and white matter hyperintensities typically disproportionately affect long-range white matter connections.^71-73^ This may be because they are particularly vulnerable to injury given their need for blood supply across multiple brain regions and their higher metabolic demand.^73-75^ Small vessel disease is often associated with cardiovascular risk factors. As most stroke survivors are older and have health conditions that may have led to the stroke (e.g. high blood pressure, diabetes, etc.), it follows that many will present with small vessel disease. Prior literature has suggested that the presence of small vessel disease (including leukoaraiosis, lacunar infarct, etc.) is associated with poorer recovery from aphasia,^68,76,77^ therefore, small vessel disease may be prevalent in a higher proportion of those who have aphasia which persists into the chronic stage post-stroke.

Within the SVR analysis, lower (more negative) beta weights indicate importance in predicting WAB-R AQ scores. Visual inspection of our results reveals that some regions appear to be more important than others in predicting behaviour in all four models: i.e. posterior superior and middle temporal gyri, posterior insula, IFG pars opercularis and the SLF. Thus, these regions, many of which are often implicated in dual stream models of language^14,78^ may represent ‘core’ regions necessary for language production and/or comprehension. For example, the motor-speech-driven dorsal stream incorporates IFG pars opercularis (which forms the posterior portion of Broca’s area), the posterior insula and SLF, and lesions in these regions have previously been associated with phonological or speech fluency deficits.^14^ On the other hand, pMTG is considered a hub within the language network^13^ and is part of the lexical-semantic ventral stream; damage to this stream is associated with auditory comprehension deficits.^79^ This study confirms the critical role of these regions in language, and as WAB-R AQ is a composite score, it is unsurprising that regions from both dorsal and ventral streams are implicated. There is often progressive degeneration of white matter integrity following stroke, and this may be more notable in the later stages of recovery. This is highlighted by the relative importance of the SLF compared to other ROIs for predicting WAB-R AQ in the chronic participants, suggesting that the integrity of this white matter tract is particularly relevant for aphasia severity in the later stages of recovery. It should be noted that despite having clinical relevance, WAB-R AQ may not be a true dimensional measure of symptoms therefore future studies could investigate specific symptomology in more detail as our theoretical and behavioural understanding of language dysfunction in stroke improves.

### Limitations

There are some potential limitations to this study. One was the cross-sectional rather than longitudinal design; participants in the acute and chronic stages were recruited at different sites by different experimenters, and lesions were drawn by different neurologists on different scan types. Out-of-sample prediction within the acute or chronic stage will likely yield lower predictive validity due to unmodelled sources of covariance (e.g. due to scanner differences, specific regional demographics etc.). Ideally, a controlled study would utilize the same patients with the same testing conditions. One factor in particular that may also be different is participant motivation: those in the chronic phase may have actively sought out studies to be involved in, whereas those in the acute phase were recruited in the hospital soon after experiencing a stroke. Participants in the acute phase also have smaller lesions and less variable behavioural scores. This is likely due to the fact that those with large lesions (and likely lower language scores) are sometimes untestable due to a reduced level of consciousness. However, the ability to predict chronic behaviour using an acute model, despite these differences, highlights the robustness of these results. This is critical for clinical practice where it is unlikely that hospitalized patients receive exactly the same testing conditions as those in chronic aphasia studies. However, future studies utilizing a longitudinal design may also consider other clinically relevant variables such as age, education or initial aphasia severity alongside lesion information when predicting behavioural outcomes.

Another limitation is that we recruited participants with both ischaemic and haemorrhagic stroke in the chronic phase, which may be problematic, as the brain regions damaged by, and the recovery patterns observed in, these differing stroke types may be different.^80-82^ Future studies may want to conduct separate analyses for these stroke types to account for potential confounds.

Finally, we would note that there are also some individuals in both leave-one-out analyses where the model is not accurately capturing their behaviour from the lesion (i.e. poor correlation between actual and predicted behaviour). This is in line with previous research highlighting that although the lesion is a significant determinant of aphasia type and severity,^61-65^ other factors also play a role, for example, age,^25,83-85^ general health^25,86^ and structural and functional connectivity.^87,88^ However, overall, these results show that information about the lesion alone gives a good indication of behavioural deficit at multiple timepoints.

## Conclusions

Our findings suggest that lesion-to-behaviour predictions were almost equivalent when comparing within stroke stage (acute, chronic) versus across stroke stage and do not appear to get worse across the acute–chronic or chronic–acute boundaries, despite different distributions of lesion profiles and aphasia severity between the two groups. Clinically, this is important as models based on one timepoint may also give us good insight into behaviour at other stages. Predictions of acute behaviour yielded higher correlations than those of chronic behaviour. It is possible that the acute lesion/perfusion pattern more accurately captures the critical changes in blood flow in the underlying vascular territories and supports the idea that the acute stage may capture the relationship between brain and behaviour more accurately. Critical brain regions in acute and chronic patients may provide us with a further understanding of recovery patterns and regions likely to have further degeneration. However, this should be tested more formally in longitudinal studies to understand the role of other factors such as age and overall health across the acute and chronic phases.

## Supplementary Material

fcad014_Supplementary_Data

## Data Availability

The data and analysis scripts that support the findings of this study are available upon reasonable request from the corresponding author, although restrictions may apply to adhere to participant consent and anonymity. Scripts used for the preprocessing of the data are available at https://github.com/neurolabusc/nii_preprocess and for the analysis of the data are available at https://github.com/grigori-yourganov/cross-predict.

## Abbreviations

ASHA =: American Speech and Language Association
IFG =: inferior temporal gyrus
IFOF =: inferior fronto-occipital fasciculus
JHU =: Johns Hopkins University
MOG =: middle occipital gyrus
MTG =: middle temporal gyrus
MP-RAGE =: magnetization-prepared rapid gradient-echo
pMTG =: posterior middle temporal gyrus
pSTG =: posterior superior temporal gyrus
RLSM =: region-based lesion symptom mapping
ROI =: region of interest
SD =: standard deviation
SLF =: superior longitudinal fasciculus
SLP =: speech-language pathologist
SMG =: supramarginal gyrus
SPACE =: Sampling Perfection with Application optimized Contrasts using different flip angle Evolution
STG =: superior temporal gyrus
UF =: uncinate fasciculus
WAB-R =: Western Aphasia Battery-Revised
WAB-R AQ =: Western Aphasia Battery-Revised Aphasia Quotient
VLSM =: voxel-based lesion-symptom mapping
