# Supplementary material for: Comparing the brain–behaviour relationship in acute and chronic stroke aphasia
Source: Brain Commun. 2023 Mar 29;5(2):fcad014. doi: 10.1093/braincomms/fcad014 (PMC10088484; doi:10.1093/braincomms/fcad014)
Supplement: fcad014_Supplementary_Data [file fcad014_supplementary_data.pdf]

Supplementary table 1.

Regions of interest from the John's Hopkins University (JHU) atlas which were significantly associated with WAB-R AQ scores, and four sub scores in RLSM analyses. For each score, regions are ordered based on z-score, with lowest (most negative) first. Regions are highlighted in grey if they appeared in both acute and chronic analyses.

Behavior	Acute		Chronic	
	ROI	Z-Score	ROI	Z-Score
WAB AQ	Posterior middle temporal gyrus	-7.01	Superior longitudinal fasciculus	-6.87
	Superior temporal gyrus	-6.95	Posterior insula	-4.85
	Angular gyrus	-6.83	Postcentral gyrus	-4.68
	Posterior superior temporal gyrus	-6.66	Supramarginal gyrus	-4.51
	Superior longitudinal fasciculus	-6.12	External Capsule	-4.46
	Middle occipital gyrus	-6.12	Internal capsule (retrolenticular)	-4.45
	Supramarginal gyrus	-5.18	Precentral gyrus	-4.23
	Postcentral gyrus	-5.16	Insular	-4.20
	Posterior insula	-5.10	Superior temporal gyrus	-4.03
	Inferior occipital gyrus	-4.89	Posterior superior temporal gyrus	-4.00
	Superior temporal gyrus pole	-4.56	Inferior frontal gyrus pars opercularis	-3.85
	Posterior inferior temporal gyrus	-4.53	Angular gyrus	-3.80
	Precentral gyrus	-4.47	Posterior middle temporal gyrus	-3.72
	Middle temporal gyrus	-4.44	Superior corona radiata	-3.71
	Posterior corona radiata	-4.12	Sagittal stratum	-3.68
			Inferior temporal gyrus	-3.66
			Middle occipital gyrus	-3.66
			Anterior corona radiata	-3.61
			Posterior corona radiata	-3.58
			Inferior frontal gyrus pars triangularis	-3.47
			Inferior fronto-occipital fasciculus	-3.46
			Fornix	-3.43
			Posterior thalamic radiation	-3.40
			Superior parietal gyrus	-3.36
			Uncinate fasciculus	-3.35
			Inferior frontal gyrus pars orbitralis	-3.24
			Middle temporal gyrus	-3.22
			Posterior inferior temporal gyrus	-3.16

			Superior occipital gyrus	-3.15
			Superior temporal gyrus pole	-3.08
			Middle frontal gyrus	-3.07
			Middle frontal gyrus (dPFC)	-3.03
			Inferior occipital gyrus	-3.00
Spontaneous Speech Score	Posterior middle temporal gyrus	-6.36	Superior longitudinal fasciculus	-6.81
	Angular gyrus	-5.95	Postcentral gyrus	-5.26
	Superior temporal gyrus	-5.80	Precentral gyrus	-5.15
	Posterior superior temporal gyrus	-5.71	Posterior insula	-4.90
	Middle occipital gyrus	-5.47	External capsule	-4.87
	Superior longitudinal fasciculus	-5.33	Inferior frontal gyrus pars opercularis	-4.53
	Inferior occipital gyrus	-4.75	Supramarginal gyrus	-4.39
	Posterior insula	-4.42	Superior corona radiata	-4.38
	Supramarginal gyrus	-4.41	Insular	-4.36
	Postcentral gyrus	-4.39	Internal capsule (retrolenticular)	-4.27
	Precentral gyrus	-4.24	Inferior frontal gyrus pars triangularis	-3.80
	Pole of superior temporal gyrus	-4.22	Anterior corona radiata	-3.62
	Superior corona radiata	-4.13	Middle frontal gyrus	-3.59
			Posterior corona radiata	-3.48
			Inferior fronto-occipital fasciculus	-3.48
			Uncinate fasciculus	-3.37
			Superior parietal gyrus	-3.36
			Superior temporal gyrus	-3.27
			Inferior frontal gyrus pars orbitralis	-3.15
			Angular gyrus	-3.13
			Middle frontal gyrus (dPFC)	-3.01
			Fornix	-2.98
Auditory Verbal Comprehension Score			Superior longitudinal fasciculus	-5.99
	Posterior middle temporal gyrus	-7.32	Posterior superior temporal gyrus	-5.15
	Angular gyrus	6.93	Posterior middle temporal gyrus	-5.00
	Posterior superior temporal gyrus	-6.30	Superior temporal gyrus	-4.82
	Middle occipital gyrus	-6.21	Posterior insula	-4.69
	Superior temporal gyrus	-6.00	Middle occipital gyrus	-4.53
	Superior longitudinal fasciculus	-5.38	Posterior thalamic radiation	-4.53
	Posterior inferior temporal gyrus	-5.22	Sagittal stratum	-4.36
	Inferior occipital gyrus	-4.90	Middle temporal gyrus	-4.30
	Middle temporal gyrus	-4.81	Angular gyrus	-4.24
	Supramarginal gyrus	-4.44	Superior occipital gyrus	-4.19

			Supramarginal gyrus	-4.16
			Internal capsule (retrolenticular)	-4.14
			Posterior inferior temporal gyrus	-4.02
			Inferior temporal gyrus	-3.98
			Inferior occipital gyrus	-3.85
			External capsule	-3.79
			Insular	-3.55
			Superior longitudinal fasciculus	-6.61
Repetition Score	Angular gyrus	-7.55	Posterior insula	-4.90
	Posterior middle temporal gyrus	-7.35	Supramarginal gyrus	-4.83
	Posterior superior temporal gyrus	-7.30	Posterior superior temporal gyrus	-4.64
	Superior temporal gyrus	-6.95	Superior temporal gyrus	-4.59
	Middle occipital gyrus	-6.57	Postcentral gyrus	-4.32
	Superior longitudinal fasciculus	-6.40	Insular	-4.26
	Supramarginal gyrus	-5.44	Posterior middle temporal gyrus	-4.20
	Inferior occipital gyrus	-5.09	Internal capsule (retrolenticular)	-4.19
	Postcentral gyrus	-4.84	External capsule	-4.15
	Posterior insula	-4.79	Angular gyrus	-3.95
	Posterior inferior temporal gyrus	-4.77	Precentral gyrus	-3.87
	Middle temporal gyrus	-4.63	Inferior frontal gyrus pars opercularis	-3.76
			Superior temporal gyrus pole	-3.74
			Inferior temporal gyrus	-3.71
			Fornix	-3.63
			Inferior frontal gyrus pars orbitalis	-3.62
			Sagittal stratum	-3.59
			Middle temporal gyrus	-3.57
			Anterior corona radiata	-3.56
			Inferior fronto-occipital fasciculus	-3.54
			Uncinate fasciculus	-3.44
			Middle occipital gyrus	-3.29
			Lateral fronto-orbital gyrus	-3.20
			Superior corona radiata	-3.14
			Middle temporal gyrus pole	-3.14
			Posterior thalamic radiation	-3.08
			Parahippocampal gyrus	-3.01
			Superior longitudinal fasciculus	-5.73
Naming & Word Finding Score	Superior temporal gyrus	-6.89	Middle occipital gyrus	-4.28
	Posterior superior temporal gyrus	-6.41	Sagittal stratum	-4.02
	Angular gyrus	-6.20	Posterior thalamic radiation	-3.99

	Posterior middle temporal gyrus	-6.17	Internal capsule (retrolenticular)	-3.88
	Superior longitudinal fasciculus	-5.77	Superior occipital gyrus	-3.75
	Posterior insula	-5.37	Inferior occipital gyrus	-3.73
	Middle occipital gyrus	-5.37	Posterior middle temporal gyrus	-3.67
	Postcentral gyrus	-5.35	Postcentral gyrus	-3.64
	Supramarginal gyrus	-5.25	Posterior inferior temporal gyrus	-3.64
	Precentral gyrus	-4.79	Inferior temporal gyrus	-3.53
	Superior temporal gyrus pole	-4.41	Posterior insula	-3.45
	Inferior occipital gyrus	-4.29	Angular gyrus	-3.41
	Middle temporal gyrus	-3.90	Anterior corona radiata	-3.35
	External capsule	-3.84	Fornix	-3.35
			Supramarginal gyrus	-3.33
			Fusiform gyrus	-3.32
			Posterior superior temporal gyrus	-3.28
			External capsule	-3.27
			Posterior corona radiata	-3.22
			Superior temporal gyrus	-3.17
			Superior parietal gyrus	-3.12
			Insular	-3.09
			Superior corona radiata	-3.08

Supplementary table 2.

Beta weights from the SVR analysis. As all participants had a left hemisphere lesion, only left hemisphere ROIs are reported. For analyses 3) and 4), one model was created for the training set and then applied to the test set so reported beta weights are from the single model. Analyses 1) and 2) were conducted using a leave-one-out approach, therefore 62 models were calculated for the acute set, and 108 for the chronic set. Reported beta weights for these analyses are averaged across these models. Beta weights are not Z-scored so relative magnitude is relevant, but the absolute magnitude is difficult to interpret.

**Acute Lesions Leave One Out**	-0.068755	posterior middle temporal gyrus
	-0.0682741	posterior superior temporal gyrus
	-0.0649235	posterior insula
	-0.0622786	angular gyrus
	-0.0565535	superior temporal gyrus
	-0.0514462	inferior frontal gyrus pars opercularis
	-0.0505297	supramarginal gyrus
	-0.0473826	middle occipital gyrus
	-0.0461082	Superior longitudinal fasciculus
	-0.0374429	external capsule
	-0.0355825	insular
	-0.0339229	middle frontal gyrus
	-0.0336742	precentral gyrus
	-0.0329077	Superior fronto-occipital fasciculus
	-0.0318437	tapatum
	-0.0282877	inferior occipital gyrus
	-0.0272709	middle temporal gyrus
	-0.0265065	superior corona radiata
	-0.0234701	Posterior thalamic radiation
	-0.0231206	anterior limb of internal capsule
	-0.0229774	lateral ventricle_atrium
	-0.0229067	posterior corona radiata
	-0.0223246	inferior frontal gyrus pars triangularis
	-0.02232	postcentral gyrus
	-0.0209928	Superior parietal gyrus
	-0.0207646	putamen
	-0.0188377	pole of superior temporal gyrus
	-0.0175693	lateral ventricle_occipital
	-0.0160409	caudate nucleus
	-0.0153754	superior occipital gyrus
	-0.0150602	Inferior fronto-occipital fasciculus
	-0.0125063	pole of middle temporal gyrus
	-0.0124531	Uncinate fasciculus
	-0.0121333	inferior frontal gyrus pars orbitralis
	-0.0113575	posterior inferior temporal gyrus
	-0.0112828	olfactory radiation

	-0.0110137	inferior temporal gyrus
	-0.0101428	anterior corona radiata
	-0.00860253	Lenticular fasciclus
	-0.00841089	globus pallidus
	-0.00720525	lateral ventricle_frontal
	-0.00637301	Sagittal stratum
	-0.00532453	middle frontal gyrus
	-0.00466112	splenium of corpus callosum
	-0.00326272	superior frontal gyrus
	-0.00283297	hypothalamus
	-0.0021561	lateral fronto-orbital gyrus
	-0.00215559	lateral ventricle_body
	-0.00199429	superior frontal gyrus
	-0.00184521	nucleus accumbens
	-0.00136592	Anterior commissure
	-0.00131768	genu of corpus callosum
	-0.0012815	body of corpus callosum
	-0.00114935	fornix
	-0.00111179	amygdala
	-0.000765649	posterior cingulate gyrus
	-0.000706741	pre-cuneus
	-0.00063698	superior frontal gyrus
	-0.000328917	Ansa lenticularis
	-0.00023082	Fornix
	-0.0000643731	cerebral peduncle
	-0.0000623185	medial lemniscus
	-0.0000614884	nucleus innominata of mynert
	-0.0000221762	III_and_IV_ventricle|III and IV ventricle
	-0.000000103006	middle fronto-orbital gyrus
	0	gyrus rectus
	0	entorhinal area
	0	rostral anterior cingulate gyrus
	0	subcallosal anterior cingulate gyrus
	0	subgenual anterior cingulate gyrus
	0	substancia nigra
	0	corticospinal tract
	0	superior cerebellar peduncle
	0	pontine crossing tract
	0	inferior cerebellar peduncle
	0	pons
	0	medulla
	0	mammillary body
	0	optic tract
	0.00027109	middle cerebellar peduncle
	0.00030437	cingulum (cingulate gyrus)
	0.00040524	dorsal anterior cingulate gyrus
	0.00050284	midbrain

	0.00134854	cerebellum
	0.00154587	lateral ventricle temporal
	0.00169547	hippocampus
	0.00186935	red nucleus
	0.0020813	fusiform gyrus
	0.00235472	parahippocampal gyrus
	0.0057495	thalamus
	0.00680183	cingulum (hippocampus)
	0.00868235	retrolenticular part of internal capsule
	0.00977086	posterior limb of internal capsule
	0.0142118	cuneus
	0.0165077	lingual gyrus
**Chronic Lesions Leave One Out**	-0.0146752	Superior longitudinal fasciculus
	-0.0120358	posterior insula
	-0.0111303	posterior superior temporal gyrus
	-0.0104379	external capsule
	-0.0101377	posterior middle temporal gyrus
	-0.00991976	supramarginal gyrus
	-0.00970266	superior corona radiata
	-0.00942861	insular
	-0.00939468	superior temporal gyrus
	-0.00903346	inferior frontal gyrus pars opercularis
	-0.00897387	angular gyrus
	-0.00893834	retrolenticular part of internal capsule
	-0.00862637	posterior corona radiata
	-0.00830796	postcentral gyrus
	-0.0075976	inferior frontal gyrus pars triangularis
	-0.00753904	precentral gyrus
	-0.00656108	anterior corona radiata
	-0.00642538	inferior frontal gyrus pars orbitralis
	-0.00641865	Inferior fronto-occipital fasciculus
	-0.00637997	Superior parietal gyrus
	-0.00637127	middle occipital gyrus
	-0.00632143	Superior fronto-occipital fasciculus
	-0.00608409	middle temporal gyrus
	-0.00565657	middle frontal gyrus (
	-0.00561538	Sagittal stratum
	-0.00538176	pole of superior temporal gyrus
	-0.00514154	posterior inferior temporal gyrus
	-0.00511995	Uncinate fasciculus
	-0.00506817	inferior temporal gyrus
	-0.00497818	Posterior thalamic radiation
	-0.00467269	inferior occipital gyrus
	-0.00415457	lateral fronto-orbital gyrus
	-0.00414128	posterior limb of internal capsule
	-0.00399123	middle frontal gyrus
	-0.00392279	pole of middle temporal gyrus
	-0.00375421	superior occipital gyrus
	-0.00288676	putamen

	-0.00261206	fornix
	-0.00222743	amygdala
	-0.00217829	ateral ventricle_temporal
	-0.00198295	superior frontal gyrus
	-0.0019556	anterior limb of internal capsule
	-0.00189501	middle fronto-orbital gyrus
	-0.00187555	fusiform gyrus
	-0.00181913	superior frontal gyrus
	-0.00175309	lateral ventricle_occipital
	-0.00154821	nucleus innominata of mynert
	-0.00151186	superior frontal gyrus
	-0.00150727	globus pallidus
	-0.00145727	Ansa lenticularis
	-0.00124736	genu of corpus callosum
	-0.00117596	cuneus
	-0.00099044	tapatum
	-0.000941519	thalamus
	-0.000941376	parahippocampal gyrus
	-0.000876987	gyrus rectus
	-0.00080381	hippocampus
	-0.000776957	dorsal anterior cingulate gyrus
	-0.000693705	entorhinal area
	-0.000654922	pre-cuneus
	-0.000486124	body of corpus callosum
	-0.000429533	rostral anterior cingulate gyrus
	-0.000398561	lingual gyrus
	-0.000370274	caudate nucleus
	-0.000350514	subcallosal anterior cingulate gyrus
	-0.000331772	lateral ventricle_atrium
	-0.000306276	cingulum (cingulate gyrus)
	-0.00030429	posterior cingulate gyrus
	-0.000235334	lateral ventricle_frontal
	-0.000218088	splenium of corpus callosum
	-0.000088008	cerebral peduncle
	-0.0000565061	optic tract
	-0.0000523999	substancia nigra
	-0.0000503317	cingulum (hippocampus)
	-0.0000498675	cerebellum
	-0.0000441999	midbrain
	-0.00000276744	medial lemniscus
	-0.00000276744	medial lemniscus
	0	corticospinal tract
	0	superior cerebellar peduncle
	0	middle cerebellar peduncle
	0	pontine crossing tract
	0	inferior cerebellar peduncle
	0	pons
	0	medulla
	0	Fornix (column and body)
	0.0000341	lateral ventricle body

	0.0000387	substancia nigra
	0.0000863	splenium of corpus callosum
	0.00013535	III and IV ventricle
	0.00016375	posterior cingulate gyrus
	0.00020293	cerebral peduncle
	0.00031288	pre-cuneus
	0.0004062	red nucleus
	0.00047009	body of corpus callosum
	0.00072524	rostral anterior cingulate gyrus
	0.00080141	optic tract
	0.00080421	Ansa lenticularis
	0.00088844	mammillary body
	0.00104426	cingulum (cingulate gyrus)
	0.00113196	nucleus accumbens
	0.00140052	hypothalamus
	0.00154592	subgenual anterior cingulate gyrus
	0.00209774	Anterior commissure
	0.00303977	olfactory radiation
	0.00520642	Lenticular fasciclus
**Predicting Acute Lesions from a Chronic Model**	-0.0280744	Superior longitudinal fasciculus
	-0.0189721	posterior insula
	-0.0188087	external capsule
	-0.0181443	posterior middle temporal gyrus
	-0.0178071	posterior superior temporal gyrus
	-0.0166857	retrolenticular part of internal capsule
	-0.0165051	superior corona radiata
	-0.0151468	inferior frontal gyrus pars opercularis
	-0.0143698	angular gyrus
	-0.0141013	insular
	-0.0140429	supramarginal gyrus
	-0.0138952	posterior corona radiata
	-0.0137597	middle occipital gyrus
	-0.0135423	postcentral gyrus
	-0.0130667	superior temporal gyrus
	-0.0126499	precentral gyrus
	-0.0116618	Superior fronto-occipital fasciculus
	-0.0104868	inferior frontal gyrus pars triangularis
	-0.0103399	posterior inferior temporal gyrus
	-0.010081	Posterior thalamic radiation
	-0.00985356	middle frontal gyrus (posterior segment)
	-0.00949418	Sagittal stratum
	-0.00904735	inferior occipital gyrus
	-0.00901462	Inferior fronto-occipital fasciculus
	-0.00900881	Superior parietal gyrus
	-0.00899695	inferior temporal gyrus
	-0.00884137	anterior corona radiata
	-0.00847677	middle temporal gyrus

	-0.0075279	posterior limb of internal capsule
	-0.00684459	Uncinate fasciculus
	-0.00677763	superior occipital gyrus
	-0.00608064	inferior frontal gyrus pars orbitralis
	-0.00568031	pole of superior temporal gyrus
	-0.00502691	middle frontal gyrus
	-0.00491049	pole of middle temporal gyrus
	-0.00488923	fornix
	-0.00437018	lateral fronto-orbital gyrus
	-0.00423594	fusiform gyrus
	-0.00421393	putamen
	-0.00356031	lateral ventricle_occipital
	-0.00311885	middle fronto-orbital gyrus
	-0.00296558	amygdala
	-0.00279591	cuneus
	-0.00264389	anterior limb of internal capsule
	-0.00255843	lateral ventricle_temporal
	-0.00255176	superior frontal gyrus (frontal pole)
	-0.00234488	nucleus innominata of mynert
	-0.00166797	tapatum
	-0.00164185	genu of corpus callosum
	-0.00156754	thalamus
	-0.00126597	parahippocampal gyrus
	-0.00116279	hippocampus
	-0.0011214	gyrus rectus
	-0.00108093	entorhinal area
	-0.000941877	lingual gyrus
	-0.000903305	superior frontal gyrus (prefrontal cortex)
	-0.000856326	superior frontal gyrus (posterior segment)
	-0.000676759	lateral ventricle_atrium
	-0.000602889	dorsal anterior cingulate gyrus
	-0.000491876	subcallosal anterior cingulate gyrus
	-0.000486686	globus pallidus
	-0.000380507	lateral ventricle frontal
	-0.000127005	cingulum (hippocampus)
	-0.000125834	cerebellum
	-0.000099619	midbrain
	-0.0000151126	caudate nucleus
	-0.00000698325	medial lemniscus
	0	corticospinal tract
	0	superior cerebellar peduncle
	0	middle cerebellar peduncle
	0	pontine crossing tract
	0	inferior cerebellar peduncle
	0	pons
	0	medulla
	0	Fornix (column and body)
	0.0000344	lateral ventricle body
	0.0000391	substancia nigra
	0.0004897	splenium of corpus callosum

	0.00013661	III and IV ventricle
	0.00016493	posterior cingulate gyrus
	0.00020341	cerebral peduncle
	0.00031592	pre-cuneus
	0.00041	red nucleus
	0.00047542	body of corpus callosum
	0.00067652	Ansa lenticularis
	0.00074354	rostral anterior cingulate gyrus
	0.00080052	optic tract
	0.00089674	mammillary body
	0.00106131	cingulum (cingulate gyrus)
	0.00114254	nucleus accumbens
	0.00141361	hypothalamus
	0.00156037	subgenual anterior cingulate gyrus
	0.00211735	Anterior commissure
	0.00306818	olfactory radiation
	0.0051956	Lenticular fasciclus
**Predicting Chronic Lesions from an Acute Model**	-0.0945362	posterior middle temporal gyrus
	-0.0852231	angular gyrus
	-0.0845473	posterior insula
	-0.0843379	posterior superior temporal gyrus
	-0.0801668	superior temporal gyrus
	-0.0641158	middle occipital gyrus
	-0.0533977	inferior frontal gyrus pars opercularis
	-0.0496393	middle temporal gyrus
	-0.0478913	supramarginal gyrus
	-0.047431	Superior longitudinal fasciculus
	-0.0410468	middle frontal gyrus (posterior segment)
	-0.0396246	precentral gyrus
	-0.0363964	external capsule
	-0.0351794	insular
	-0.0293545	Superior parietal gyrus
	-0.0286616	inferior occipital gyrus
	-0.0281247	pole of superior temporal gyrus
	-0.0278004	Superior fronto-occipital fasciculus
	-0.0263882	anterior limb of internal capsule
	-0.0258875	tapatum
	-0.0241826	inferior frontal gyrus pars triangularis
	-0.0240809	postcentral gyrus
	-0.0240327	pole of middle temporal gyrus
	-0.021942	superior corona radiata
	-0.0201008	lateral ventricle_atrium
	-0.0198222	Posterior thalamic radiation
	-0.0196731	inferior temporal gyrus
	-0.0176704	posterior inferior temporal gyrus
	-0.0176539	globus pallidus
	-0.0155566	putamen
	-0.0154666	caudate nucleus

	-0.0149276	anterior corona radiata
	-0.0148812	superior occipital gyrus
	-0.0132422	Lenticular fasciculus
	-0.0127929	inferior frontal gyrus pars orbitralis
	-0.0107869	posterior corona radiata
	-0.00795819	olfactory radiation
	-0.00773534	lateral ventricle_frontal
	-0.00695809	Inferior fronto-occipital fasciculus
	-0.00657992	lateral ventricle_occipital
	-0.006338	superior frontal gyrus (posterior segment)
	-0.00598686	middle frontal gyrus (dorsal prefrontal cortex)
	-0.00405396	superior frontal gyrus (prefrontal cortex)
	-0.00300095	Anterior commissure
	-0.00294272	splenium of corpus callosum
	-0.00267857	genu of corpus callosum
	-0.00220419	fornix (cres)
	-0.00208716	nucleus accumbens
	-0.00204912	lateral fronto-orbital gyrus
	-0.00201394	Uncinate fasciculus
	-0.00172885	hypothalamus
	-0.00129484	superior frontal gyrus (frontal pole)
	-0.000831962	amygdala
	-0.000662234	body of corpus callosum
	-0.000141459	cerebral peduncle
	-0.000136915	medial lemniscus
	-0.0000375	nucleus innominata of mynert
	-0.0000135	III and IV ventricle
	0	middle fronto-orbital gyrus
	0	gyrus rectus
	0	entorhinal area
	0	rostral anterior cingulate gyrus
	0	subcallosal anterior cingulate gyrus
	0	subgenual anterior cingulate gyrus
	0	substancia nigra
	0	corticospinal tract
	0	superior cerebellar peduncle
	0	pontine crossing tract
	0	inferior cerebellar peduncle
	0	pons
	0	medulla
	0	Fornix (column and body)
	0	mammillary body

	0	optic tract
	0.000164019	middle cerebellar peduncle
	0.000197829	pre-cuneus
	0.00070303	posterior cingulate gyrus
	0.000815917	cerebellum
	0.000820434	dorsal anterior cingulate gyrus
	0.00100951	cingulum (cingulate gyrus)
	0.00102218	midbrain
	0.0013123	Sagittal stratum
	0.00166829	fusiform gyrus
	0.00294147	lateral ventricle temporal
	0.0038	red nucleus
	0.00460149	Ansa lenticularis
	0.00499099	hippocampus
	0.00517813	lateral ventricle body
	0.00577329	parahippocampal gyrus
	0.0092758	cingulum (hippocampus)
	0.0111488	thalamus
	0.0188437	lingual gyrus
	0.0203388	cuneus
	0.0235638	posterior limb of internal capsule
	0.0330823	retrolenticular part of internal capsule
